# Data on sulforaphane treatment mediated suppression of autoreactive, inflammatory M1 macrophages

**DOI:** 10.1016/j.dib.2016.03.105

**Published:** 2016-04-25

**Authors:** Sanjima Pal, V. Badireenath Konkimalla

**Affiliations:** School of Biological Sciences, National Institute of Science Education and Research (NISER), PO Bhimpur-Padanpur, Via-Jatni, Khurda 752050, India

## Abstract

Any chronic, inflammatory, autoimmune disease (e.g. arthritis) associated pathogenesis directs uncontrolled accumulation of both soluble forms of collagens in the synovial fluids and M1 macrophages around inflamed tissues. Despite of few studies demonstrating efficiency of Sulforaphane (SFN) in suppressing arthritis associated collagen restricted T cells or fibroblasts, its effects on macrophage polarity and plasticity are less understood. Recently, we reported regulation of phenotypic and functional switching by SFN in induced and spontaneously differentiating human monocytes [1]. Here, flow cytometry, western blot and ELISA derived data demonstrated that SFN inhibited *in vitro* inflammatory responses developed by soluble human collagens (I–IV) induced auto-reactive M1 type monocyte/macrophage model.

## **Specifications Table**

**Table:** 

Subject area	*Biology*
More specific subject area	*Immune regulation by phytochemical*
Type of data	*Image (western blot), bar-graphs, flow cytometry derived dot plots and histograms*
How data was acquired	*Protein Electrophoresis apparatus (Hoefer, USA), Semi-dry electro-blotter (Bio-rad, USA), Gel Doc-XRS (Biorad, USA), FACSCalibur^TM^ (BD Biosciences, USA), Microplate reader (Biorad, USA)*
Data format	*Filtered, analyzed*
Experimental factors	*Human monocytic cell line,THP1, Phorbol 12-myristate 13-acetate (PMA), Lipopolysaccharides (LPS), Interferon gamma (IFNγ), Sulforaphane (SFN), Human collagens (types I-IV)*
Experimental features	*All analysis was performed using Microsoft Excel or GraphPad software. The results were expressed as either mean±SD or mean±SEM.*
Data source location	*National Institute of Science Education and Research (NISER), Bhubaneswar, India*
Data accessibility	*Data are presented in this article.*

## **Value of the data**

• These *in vitro* data provide the information on soluble form of human collagen induced functional M1 polarization in PMA differentiating THP1 monocytes.
• These data indicate the efficiency of SFN to bring about phenotypic and functional changes in auto-reactive M1 cells.
• Data indicates, SFN suppresses auto-reactive M1 macrophages (soluble collagen induced) by targeting surface CD36, endogenous COX-2, and both inflammatory (IL12p70) and anti-inflammatory (IL10) cytokines.

## 1 Data

After comparing a few features of conventional inflammatory M1 cells [2], [3], [4], the western blot (see Fig. S1a) and ELISA data (see Fig. S1b) indicate that soluble human collagen-I polarized THP1 monocytes differentiation towards M1 type. Other western blot, FACS profile and quantitative data obtained from sandwich ELISA have demonstrated that SFN can decrease M1 biomarkers and increase M2 biomarkers in soluble human collagen induced M1 cells, another sensitive biomarker of chronic arthritic diseases [5], [6], [7] (see Figs. S2–S5).

## 2 Experimental design, materials and methods

### 2.1 Reagents and antibodies

Buffers and cell culture media such as 10× PBS (#ML023), DMEM (#AL007A), RPMI (#AL162S), penicillin–streptomycin (Cat no.: A001), Fetal Bovine Serum (#RM9970) were purchased from HIMEDIA. Collagen type I (C5483), IFNγ (SRP3058) from human origins, lipopolysaccharide from *E. coli* 0127:B8 (L3129) as well as chemicals, PMA (P8139) Tris–HCl (#T5941), Trizma base (#T1503), sodium chloride (#S3014) and methanol (#154903) were procured from Sigma, USA. Several other chemicals such as L-Sulforaphane [0219378210], DMSO [196055] and protease inhibitor cocktails [158837] were obtained from MP biomedicals, USA. Collagen type II (CC052), III (CC054), and IV (CC076) were purchased from Millipore, USA.

For Western blotting, specific, anti-human primary antibodies raised against COX-2 (#4842), β-actin (#4970) were obtained from cell signaling technology, USA. Flow cytometry based, fluorophore conjugated monoclonal antibodies such as mouse anti-human CCR7 (CD197) antibody conjugated to FITC (560548), mouse anti-human CD36 conjugated to APC (550956) and mouse anti-human CD206 conjugated to FITC (551135) were obtained from BD biosciences, USA.

### 2.2 Generation of conventional M1, auto-reactive M1 and immune-suppressive (M2) macrophages

**Scheme 1:**
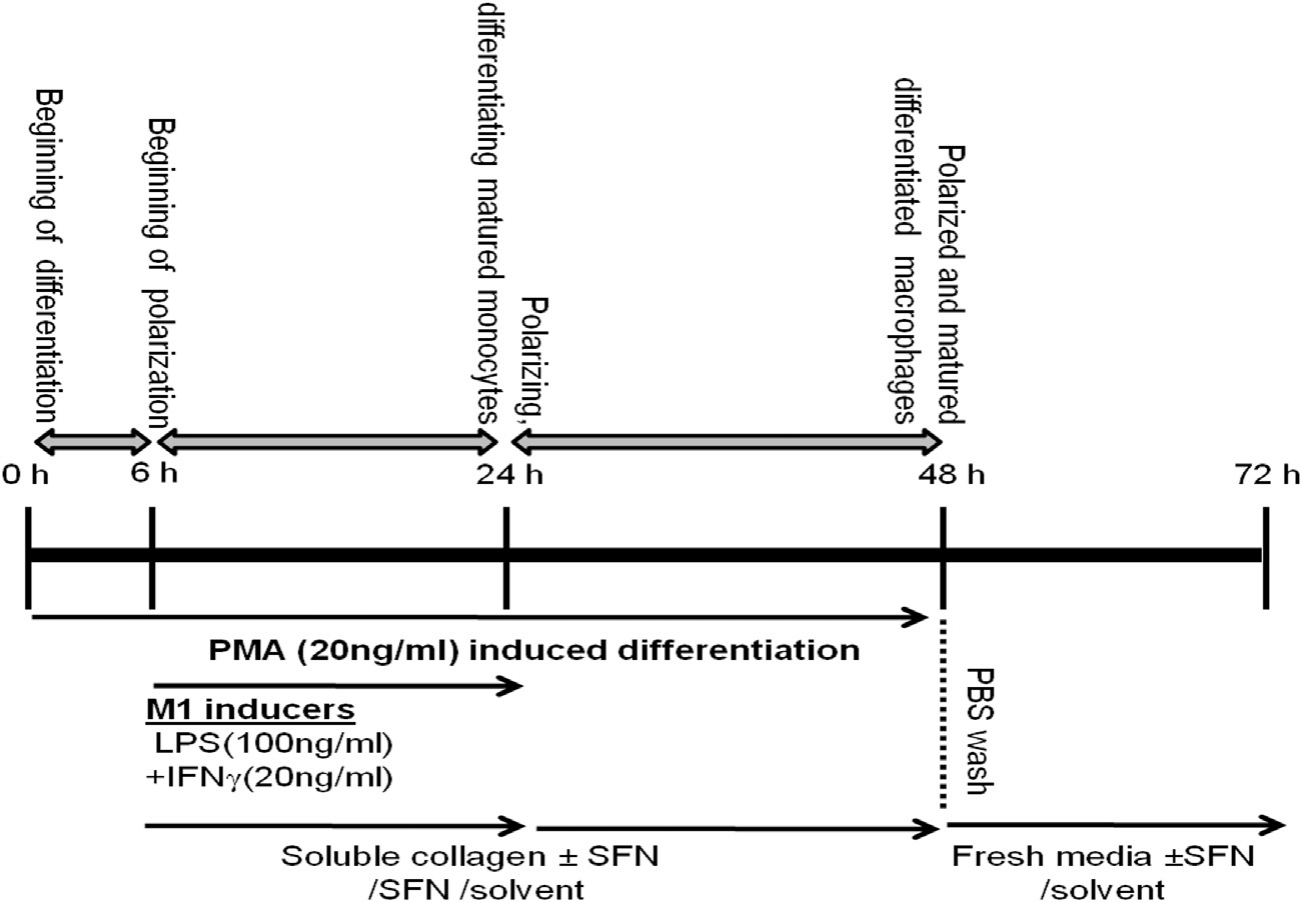
Schematic illustration of experimental plans using human monocytic cell line, THP1.

Human monocytic cell line, THP1 was selected for the experiments due to its ability to differentiate into M1 or M2 type macrophages depending on external stimuli [8], [9], [10]. About 0.5×10^6^ THP1 monocytes were treated with 20 ng/ml PMA alone for initial 6 h and then treated (in presence of PMA) with LPS (100 ng/ml) and IFNγ (20 ng/ml) together (to derive conventional inflammatory M1 cells) or 1 µg/ml soluble collagens I–IV (biomarker of chronic arthritic diseases [11], [12]) alone or with SFN alone for another 24–48 h to polarize THP1 monocytes differentiation. The work plan is described in Scheme 1.

### 2.3 Western blotting

Whole cell lysates from individual treatments were prepared in RIPA lysis buffer (150 mM NaCl, 1% Triton X-100, 0.5% sodium deoxycholate, 0.1% SDS or sodium dodecyl sulfate, 50 mM Tris buffer pH-8 and 2× protease inhibitor cocktail). Protein concentrations were measured using the Bradford reagent (Bio-rad #500-0205) and equal amount of total protein per sample was separated by SDS-PAGE and transferred onto PVDF membranes (IPVH15150, 0.44 µm, Millipore, USA) or (#162-0177, 0.22 µm, Bio-rad, USA) by semi-dry electro-blotting (Bio-rad,USA). After blocking (5% skimmed milk), proteins detected by incubating the blots with 1:1000 diluted rabbit anti-human monoclonal/polyclonal antibodies specific for the respective protein followed by a goat anti-rabbit antibody conjugated to horseradish peroxidase (HRP) (#7074,Cell Signaling, USA). Chemi-luminescence was generated using the enhanced chemiluminescence ECL kit (#7003, 20× LumiGLO® Reagent and 20× Peroxide, Cell Signaling, USA) following manufacturer׳s instructions and recorded under Gel Doc-XRS (Biorad,USA). β-actin was also detected in all the experimental conditions to ensure uniform protein loading.

### 2.4 Cytokine measurement

Using sandwich ELISA, M1 and M2 specific cytokine production was measured in the supernatants isolated from each experimental matured macrophages. For this purpose, BD OptEIA™ (BD biosciences) containing sets for both IL-12p70 (M1 type, Cat no. 555183) and IL-10 (M2 type, Cat no. 555157) were used as per the manufacturer instructions to quantify the cytokine levels simultaneously. The optical densities at 450 nm were recorded using Biorad plate reader and compared with a standard curve generated for each cytokine to determine the concentration in each of the experimental samples.

### 2.5 Surface markers expression

Surface marker expressions on the cells belong to each experimental set up (in presence and absence of indicated external agents) were analyzed by flow cytometry using FACS CaliburFlow Cytometer (BD Biosciences). Following treatment, the cells were harvested, washed and a single-cell suspension were prepared using ice cold PBS. Cells were then washed before and after staining with 500 µL of PBS containing 0.1% BSA and 0.02% sodium azide (FACS buffer). These cells were then labeled with specific monoclonal antibodies for 30 min in 50 µL of FACS buffer. About 10,000 events per sample were acquired and the mean fluorescence intensity (MFI) of cells staining positive for each surface protein was determined by comparing test samples with unstained, negative control samples.

## Footnotes

Appendix A Supplementary data associated with this article can be found in the online version at 10.1016/j.dib.2016.03.105.
