# Supplementary material for: Data on sulforaphane treatment mediated suppression of autoreactive, inflammatory M1 macrophages
Source: Data Brief. 2016 Apr 25;7:1560–4. doi: 10.1016/j.dib.2016.03.105 (PMC4865660; doi:10.1016/j.dib.2016.03.105)
Supplement: Supplementary file 1 — Supplementary material [file mmc1.docx]

**CONFLICT OF INTEREST**

We wish to confirm that there are no known conflicts of interest associated with this publication. We confirm that the manuscript has been read and approved by all named authors and that there are no other persons who satisfied the criteria for authorship. We understand that the Corresponding Author is the sole contact for the Editorial process (including Editorial Manager and direct communications with the office). He/she is responsible for communicating with the other authors about progress, submissions of revisions and final approval of proofs.

Dr. V Badireenath Konkimalla

(Corresponding author)
