# Supplementary figures and images for: Data on sulforaphane treatment mediated suppression of autoreactive, inflammatory M1 macrophages
Source: Data Brief. 2016 Apr 25;7:1560–4. doi: 10.1016/j.dib.2016.03.105 (PMC4865660; doi:10.1016/j.dib.2016.03.105)

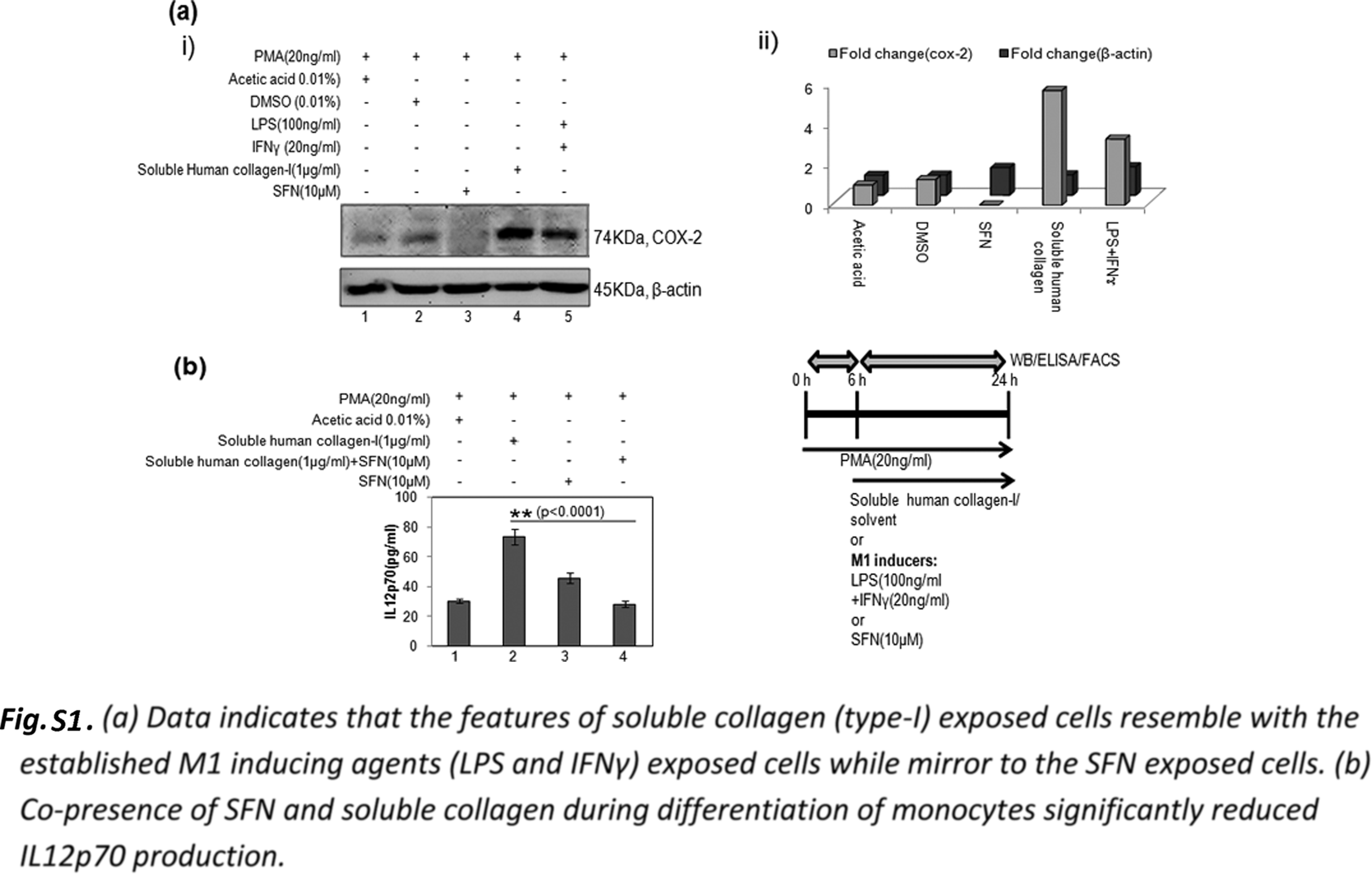

Supplement: Supplementary file 2 — Supplementary material [file mmc2.zip › Supplementary Figures TIFF/Supplementary Figure 1.tif]

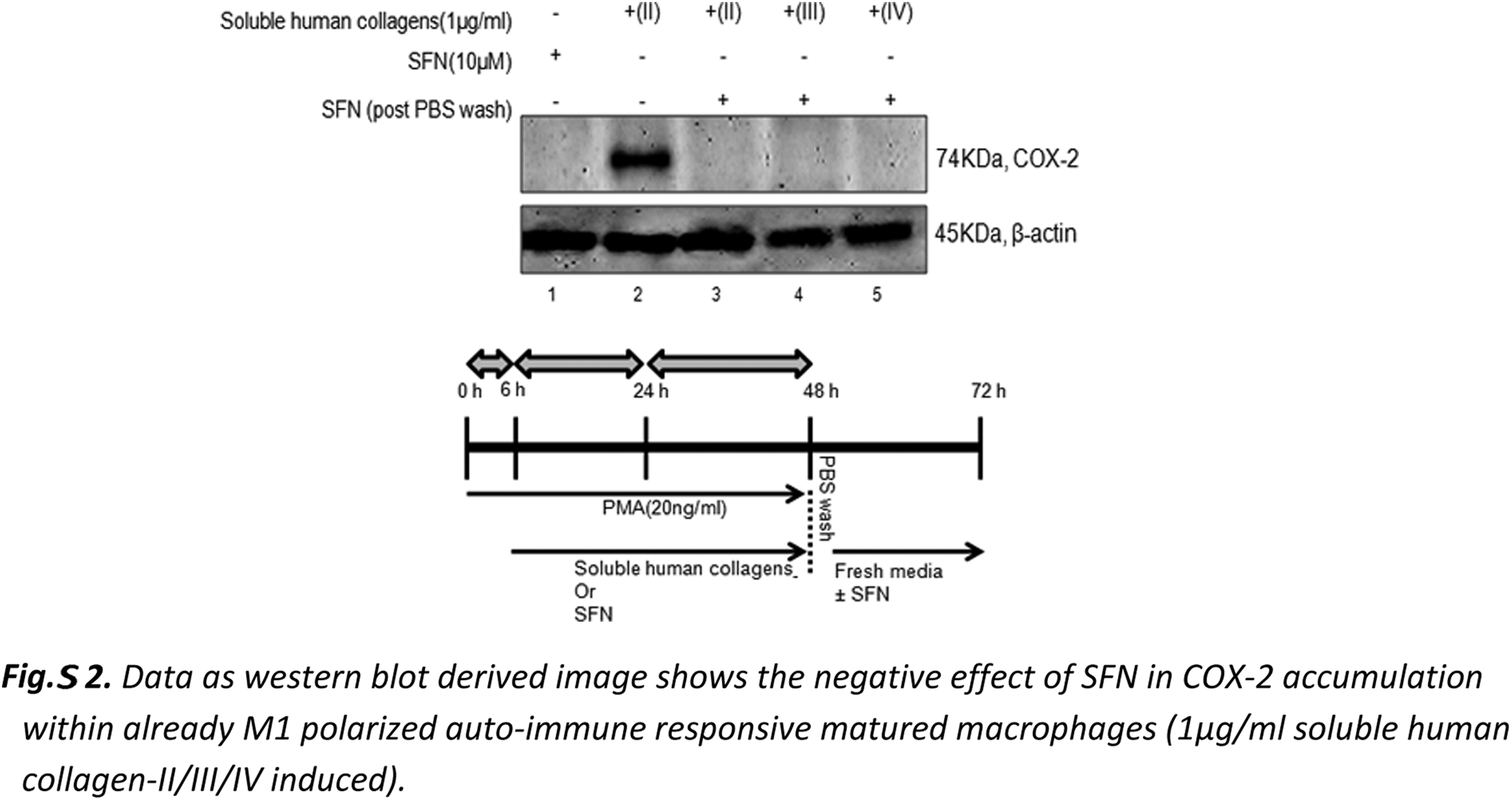

Supplement: Supplementary file 2 — Supplementary material [file mmc2.zip › Supplementary Figures TIFF/Supplementary Figure 2.tif]

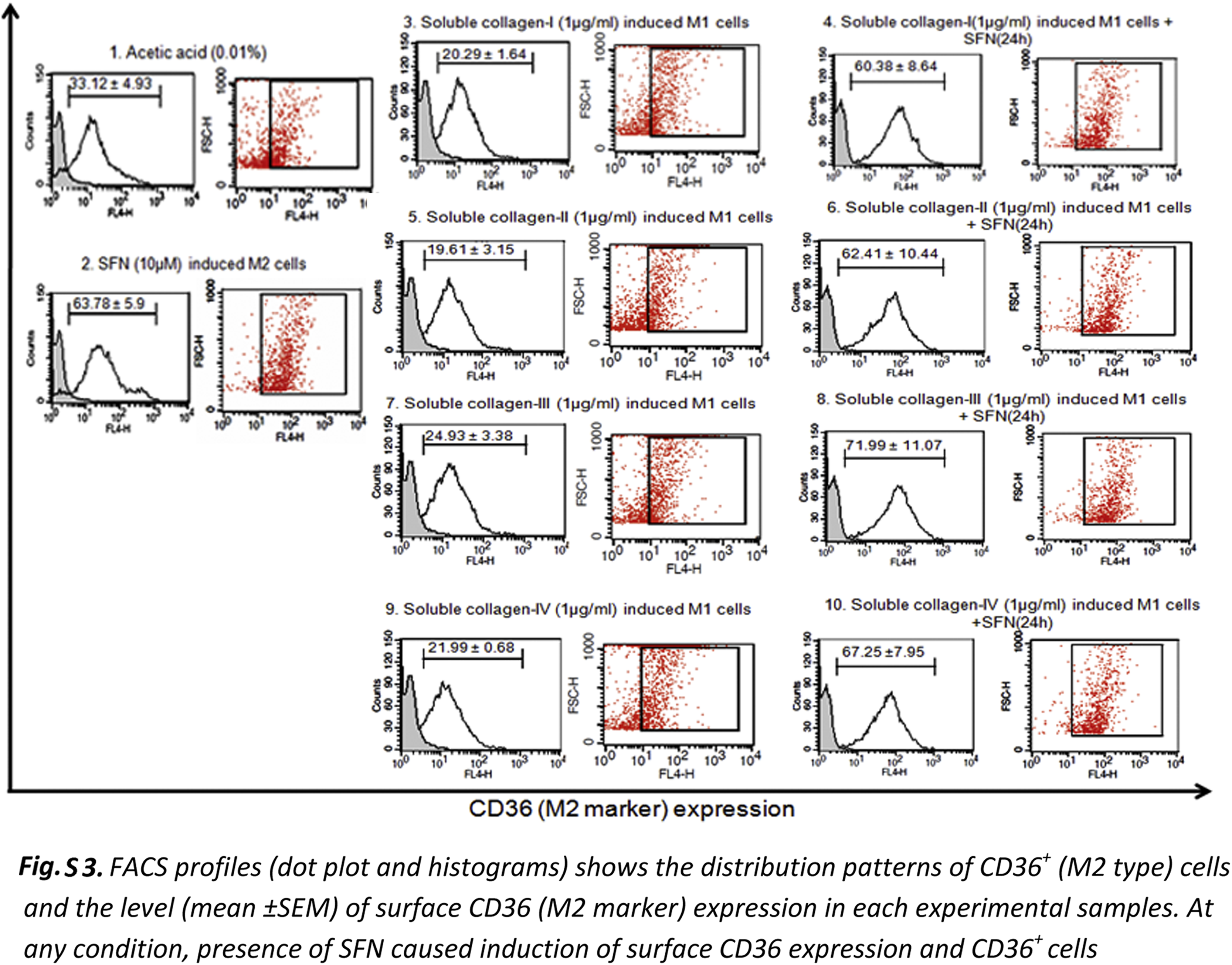

Supplement: Supplementary file 2 — Supplementary material [file mmc2.zip › Supplementary Figures TIFF/Supplementary Figure 3.tif]

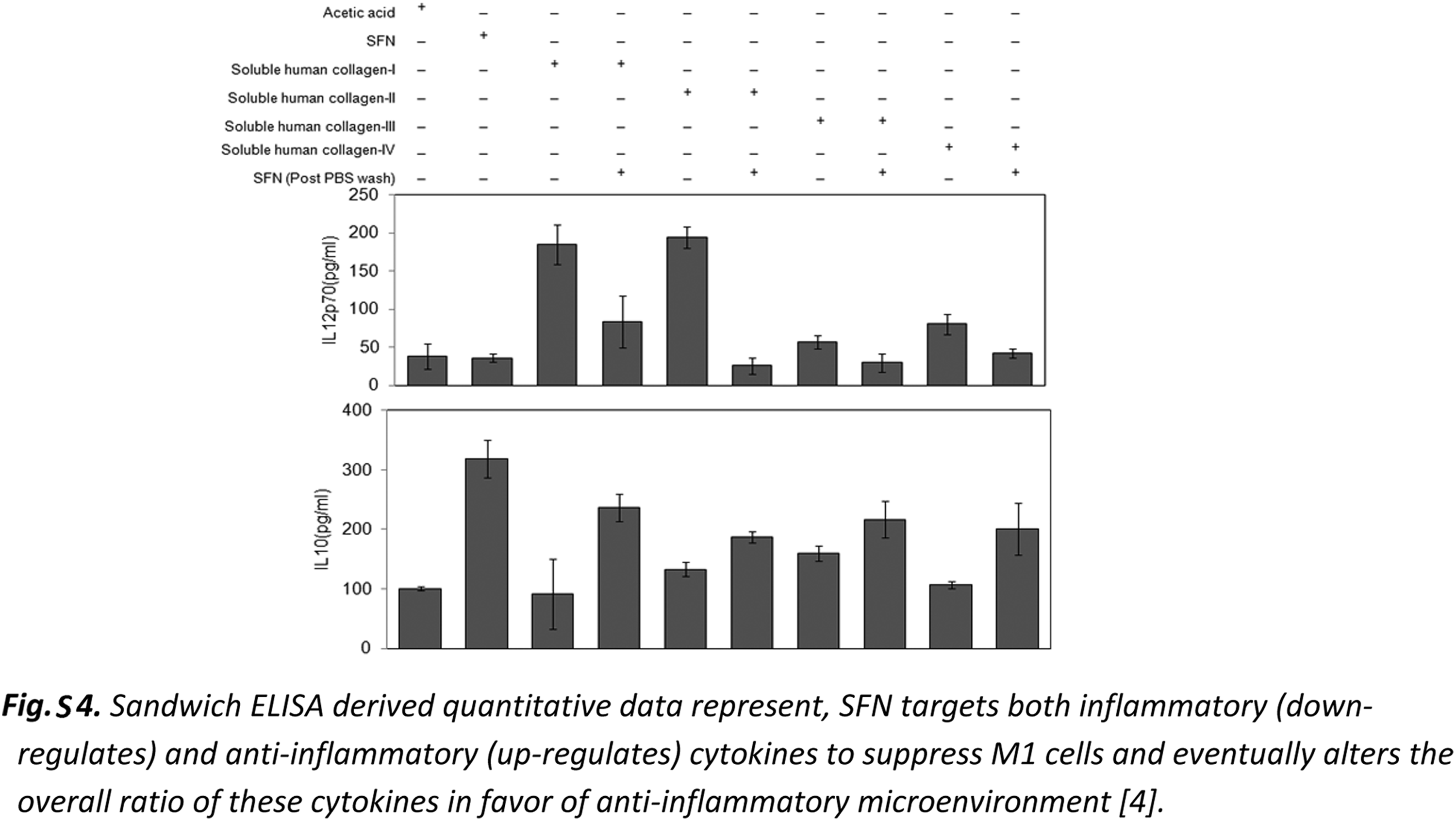

Supplement: Supplementary file 2 — Supplementary material [file mmc2.zip › Supplementary Figures TIFF/Supplementary Figure 4.tif]

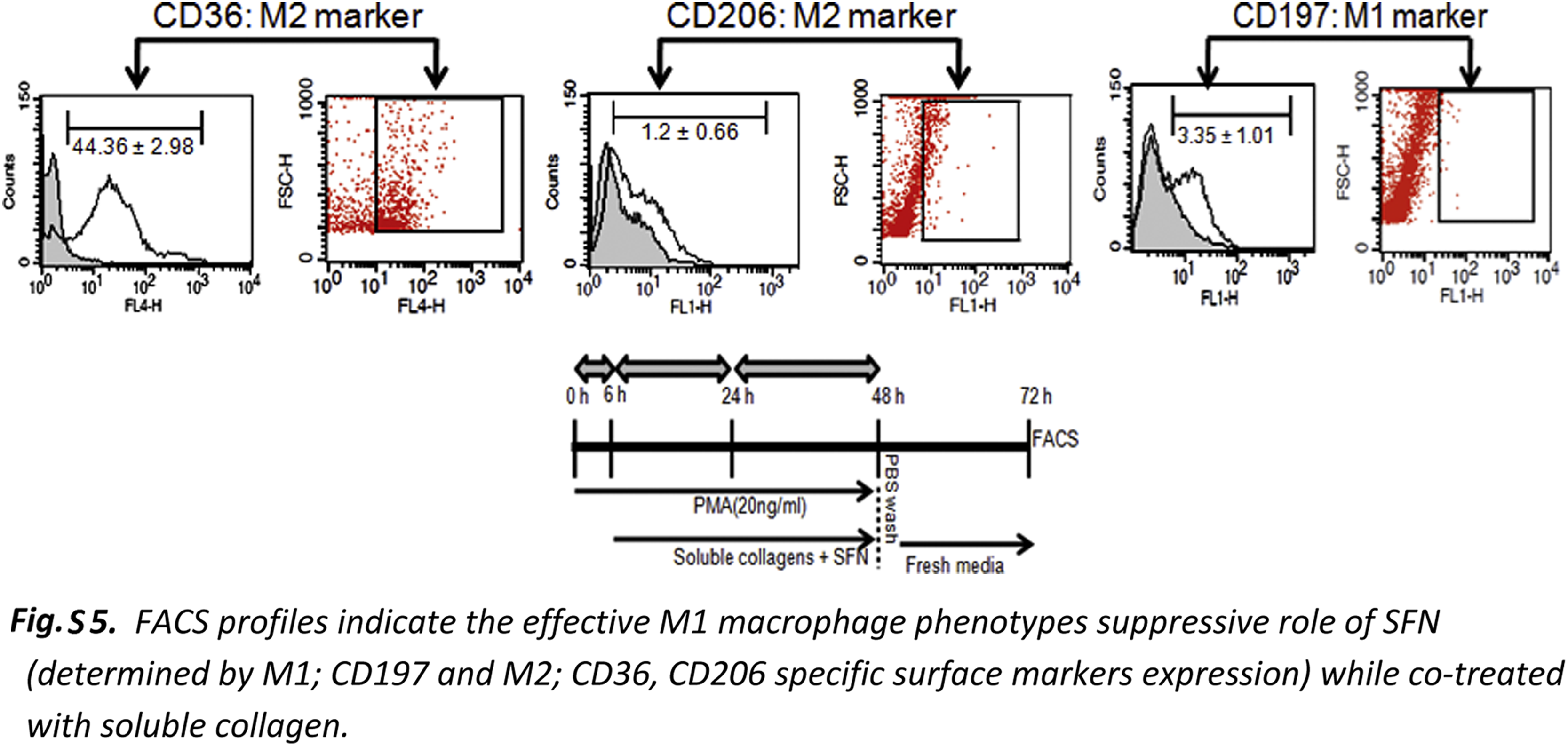

Supplement: Supplementary file 2 — Supplementary material [file mmc2.zip › Supplementary Figures TIFF/Supplementary Figure 5.tif]
